# The importance of iron in long-term survival of maintenance hemodialysis patients treated with epoetin-alfa and intravenous iron: analysis of 9.5 years of prospectively collected data

**DOI:** 10.1186/1471-2369-10-6

**Published:** 2009-02-26

**Authors:** Victor E Pollak, Jonathan A Lorch, Rakesh Shukla, Supriya Satwah

**Affiliations:** 1 MIQS Inc., 2100 Central Avenue, Suite 201, Boulder, Colorado 80301, USA; 2 The Rogosin Institute, 505 East 70th Street, New York, NY 10021, USA; 3 Department of Environmental Health, University of Cincinnati, 3223 Eden Avenue, Cincinnati, Ohio 45267- 005, USA

## Abstract

**Background:**

In patients treated by maintenance hemodialysis the relationship to survival of hemoglobin level and administered epoetin-alfa and intravenous iron is controversial. The study aim was to determine effects on patient survival of administered epoetin-alfa and intravenous iron, and of hemoglobin and variables related to iron status.

**Methods:**

The patients were 1774 treated by maintenance hemodialysis in 3 dialysis units in New York, NY from January 1998 to June, 2007. A patient-centered, coded, electronic patient record used in patient care enabled retrospective analysis of data collected prospectively. For survival analysis, patients were censored when transplanted, transferred to hemodialysis at home or elsewhere, peritoneal dialysis. Univariate Kaplan-Meier analysis was followed by multivariate analysis with Cox's regression, using as variables age, race, gender, major co-morbid conditions, epoetin-alfa and intravenous iron administered, and 15 laboratory tests.

**Results:**

Median age was 59 years, epoetin-alfa (interquartile range) 18,162 (12,099, 27,741) units/week, intravenous iron 301 (202, 455) mg/month, survival 789 (354, 1489) days. Median hemoglobin was 116 (110, 120)g/L, transferrin saturation 29.7 (24.9, 35.1)%, serum ferritin 526 (247, 833) μg/L, serum albumin 39.0 (36.3, 41.5) g/L. Survival was better the higher the hemoglobin, best with > 120 g/L. Epoetin-alfa effect on survival was weak but had statistically significant interaction with intravenous iron. For intravenous iron, survival was best with 1–202 mg/month, slightly worse with 202–455 mg/month; it was worst with no intravenous iron, only slightly better with > 455 mg/month. Survival was worst with transferrin saturation ≤ 16%, serum ferritin ≤ 100 μg/L, best with transferrin saturation > 25%, serum ferritin > 600 μg/L The effects of each of hemoglobin, intravenous iron, transferrin saturation, and serum ferritin on survival were independently significant and not mediated by other predictors in the model.

**Conclusion:**

Long term survival of maintenance hemodialysis patients was favorably affected by a relatively high hemoglobin level, by moderate intravenous iron administration, and by indicators of iron sufficiency. It was unfavorably influenced by a low hemoglobin level, and by indicators of iron deficiency.

## Background

Deficiencies of erythropoietin and iron play a role in genesis of the anemia of Chronic Kidney Disease (CKD) and End Stage Renal Disease (ESRD) patients treated by hemodialysis (HD). Both are correctible. Although treatment with epoetin-alfa (EPO) and newer erythrocyte stimulating agents (ESA) has mitigated its severity, some recent studies have reported adverse outcomes of effective anemia correction with EPO and other ESAs [1-3].

Iron deficiency anemia is prevalent in 2–8% of adult women [4], latent iron deficiency (i.e., without anemia) may be twice as frequent [5]. In a complex, stratified random US population sample of 15,387 subjects, anemia (hemoglobin < 110 g/L in women, < 120 g/L in men) was found in 569 (3.9%), in 179 of whom creatinine clearance was < 50 ml/min. Of these, < 33% were iron sufficient (TSAT ≥ 20%, serum ferritin ≥ 100 μg/L) [6]. Thus iron deficiency and iron deficiency anemia are highly prevalent in renal insufficiency even before dialysis is required. Recent studies have confirmed this [2,7-11], and intravenous (IV) iron replenishment slowed deterioration of renal function in CKD [7,8].

Possible adverse effects of raising hemoglobin (Hb) concentration to > 120 g/L on patient survival have attracted wide attention recently, and have resulted in warnings about the practice. The importance of adequate iron availability was stressed in the earliest EPO studies in HD patients [12], yet few reports on the effects of EPO and newer ESAs have explicitly addressed the need of patients for adequate iron [2,3,10], and ESA effects have been tested in CKD patients at least 25–30% of whom had evidence of iron deficiency at study start before the ESA was given [2,3,10]. Also, widely publicized guidelines suggesting adverse effects of serum ferritin levels > 500–1,000 μg/L [13-16] have cautioned about IV iron usage.

We recently reported the effect of a particular patient-centered electronic patient record (EPR) on mortality in 1,790 patients treated by in-center HD in 3 dialysis units over 7–9 years [17]. That analysis was limited in at least two respects. First, patient mortality was the measured outcome; second, the effect of various prognostic factors on mortality was assessed one predictor at a time. Here, we apply univariate and multivariate methods to examine the effects of various factors, including EPO and IV iron administration, on patient survival. The data analyzed were those collected prospectively during patient care, using the EPR, from 1998 onward. We report on long-term survival of these patients in relation to anemia and its management with EPO and IV iron.

## Methods

### Patients

A total of 1790 patients was available who were treated by maintenance in-center HD in 3 dialysis units managed by The Rogosin Institute (New York, NY), affiliated with New York Presbyterian Hospital and Weill Medical College of Cornell University; 1774 were included in the present study. Because they had hemolytic anemias and persistently high serum ferritin levels in association with the primary disease, 15 patients with sickle cell anemia and sickle cell trait were excluded, as well as one with acute myeloid leukemia who had recently received a bone marrow transplant and total body irradiation. The patient characteristics were described in detail previously [17]. All started the in-center HD course between January 1, 1998 and December 31, 2006. The present study includes survival follow up until June 30, 2007.

EPO, IV iron, and other medications were prescribed by attending nephrologists using an approach guided, with assistance of an anemia management team, by then current NKF DOQI guidelines available in 2002 [14] and 2006 [15]. EPO was administered IV. Three IV iron preparations were used: iron dextran (InFed^®^), iron sucrose (Venofer^®^), and sodium ferric gluconate (Ferrlecit^®^). Clinical practice was also influenced by changing mandates from the Center for Medicare and Medicaid Services (CMS) and by publications suggesting adverse effects of high serum ferritin levels [13].

A comprehensive patient-centric, computerized record system (Disease Manager Plus™, MIQS, Inc., Boulder CO), in which data are stored in coded, extractable, form was used in patient care. Described in detail elsewhere, it enabled patient management and the present analyses [17]. Variables abstracted for analysis included:

• Demographic: age at treatment start, race; gender; primary renal disease leading to ESRD

• Co-morbid diseases present at or before treatment start: arteriosclerotic heart disease (ICD9-CM 410–414, 36.06, 36.07, 36.1), cerebrovascular disease (ICD 430–438), peripheral vascular disease (ICD 440, 441, 443, 785.4, V49.7, 84.1), malignancy (ICD 149–208), diabetes mellitus Types I (ICD 250.x1) and II (ICD 250.x0), AIDS (ICD 042), peptic ulcer disease (ICD 531–533).

• Drugs: EPO and IV iron recorded as administered during HD treatments on 430,077 and 135,595 occasions respectively. During the course of the study there were three double blind dose equivalence studies, comparing then new ESAs with EPO. In all, these involved 3,531 (0.82%) of all ESA administrations. Data from these double blind studies have been included using the appropriate dose of EPO and its assumed equivalent.

• Laboratory test results: Hb, red blood cells (RBC), mean corpuscular volume (MCV), mean corpuscular hemoglobin (MCH), mean corpuscular hemoglobin concentration (MCHC), serum iron, transferrin saturation (TSAT), total iron binding capacity (TIBC), serum calcium, serum phosphorus, calcium × phosphorus product, serum intact parathyroid hormone (iPTH), Kt/V, and serum albumin were obtained on admission and monthly thereafter. Serum ferritin was measured 3-monthly. Approximately 58,980 measurements of each set of laboratory tests were analyzed.

Patients who continued in-center HD without interruption were followed until death or study termination on June 30, 2007. Save for the 29 who returned to in-center HD within 3 months of the last in-center HD treatment, those treated subsequently in another dialysis unit, or by kidney transplantation, home HD, or peritoneal dialysis were censored at the time of their last in-center HD treatments.

### Statistical Analysis

Survival was defined from the date of the first in-center HD during the study period to death or termination of the follow-up (June 30, 2007), at which time patients still alive were treated as censored for survival analyses. Patients were also censored at the last in-center HD treatment before kidney transplantation, start of home HD, peritoneal dialysis, or transfer to another dialysis unit for in-center maintenance HD treatment.

Since each patient had numerous administered doses of EPO and IV iron, we obtained each patient's EPO dose as units per week, and IV iron dose as mg per month. This single characterization for each patient allowed us to summarize rates of administration of EPO and IV iron for each patient, and thereby assess the effects of these treatments on survival. Similarly, each hematological parameter for each patient was summarized by the mean value within that patient, thereby representing an average assessment over the duration of observation for that patient. The effects of various predictors on survival discussed herein should therefore be interpreted as "between-patient" rather than "within-patient" effects.

While EPO and IV iron dosages and hematological predictors are measured on interval scales, we examine these predictors both as continuous and as categorical variables for ease of interpretation. For Hb, serum iron, TSAT, serum ferritin, and other laboratory variables clinically relevant specific categorizations and cut-offs are used.

We employed standard regression techniques for survival data. Kaplan-Meier curves were obtained [18] for univariate analyses and, to ensure adequate consideration of confounding factors, Cox multivariate regression analysis [19]. The following co-variates were considered in the model building stage of analysis: age at treatment start, race, gender, primary renal disease leading to ESRD, presence or absence of one or more co-morbid conditions, EPO and IV iron dose administered, and hemoglobin, RBC, MCV, MCH, MCHC, serum iron, TSAT, TIBC, serum calcium, serum phosphorus, calcium × phosphorus product, iPTH, Kt/V, and serum albumin. We used a backward regression approach in multivariate set up to eliminate non-significant (p > 0.05) predictors from the model. We analyzed the EPO interaction effects on patient survival with IV iron, TSAT and serum ferritin in three models each of which contained EPO administered and one only of IV iron administered, TSAT, or serum ferritin along with all other significant predictors of survival. Thus, the above mentioned three interactions were assessed but only as two-factor interactions, and one interaction at a time with categorical characterization of the interacting variables. Only when the test for interactions was significant (P < 0.05), we looked at the pair-wise hazard ratios for assessing effects of interacting variables.

EPO was administered to 1731 patients (97.6%), TSAT values were available on 1690 patients (95.3%), and serum ferritin values on 1710 (96.4%). Certain variables which were skewed in nature were log transformed.

### Study Approval

The study was approved by the Institutional Review Board of Weill Medical College of Cornell University.

## Results

### Patients

The patient characteristics are summarized in Table 1. Mean age at study start was 59 years, 53% were male, 43% White, and 36% Black; 18% were Hispanic. The primary disease leading to renal failure was glomerulonephritis in 15%, hypertension in 24%, and diabetes mellitus Types I and II in 4% and 32% respectively. Co-morbid conditions were: arteriosclerotic heart disease in 23%. cerebral vascular disease in 8%, peripheral vascular disease in 12%, malignant disease in 9%, AIDS in 4.2%, diabetes mellitus Types I and II in 4.6 and 38% respectively, and peptic ulcer in 2.1%; 649 patients (36.6%) had none of these co-morbid conditions.

**Table 1:** Selected patient data at time of the first in-center HD treatment

		%
Patients	1774	

Age at start of first in-center HD treatment	59 ± 16.2	

Gender (male/female)	944:830	53.2:46.8

Race (White: Black: Other/Unknown)		42.7:36.3:21.0

**Primary disease leading to renal failure**		

Glomerulonephritis	259	14.6

Hypertension	427	24.1

Diabetes mellitus Type I (juvenile)	71	4

Diabetes mellitus Type II (adult onset or unspecified)	577	32.4

Other	440	24.8

**Major known co-morbid conditions at study entry**		

Arteriosclerotic heart disease	410	23.1

Cerebral vascular disease	148	8.3

Peripheral vascular disease	215	12.1

Malignant disease	161	9.1

AIDS	74	4.2

Diabetes mellitus Type I (juvenile)	82	4.62

Diabetes mellitus Type II (adult onset or unspecified)	670	37.8

Peptic ulcer disease	37	2.1

Previous kidney transplant	74	4.2

Survival duration and EPO and IV iron administered are summarized in Table 2. Median survival was 789 days (interquartile range 354 and 1489). The median EPO and IV iron administered were, respectively, 18.16 (IQR 12.10 and 27.74) 10^3 ^units/week and 301.2 (IQR 201.6 and 454.7) mg/month.

**Table 2:** Median patient survival, and median EPO and IV iron given for the total study duration

	Median	Interquartile range (25%)	Interquartile range (75%)
Survival duration (days)	789	354	1489
EPO administered per week (10^3 ^units)	18.16	12.1	27.74
IV iron administered per month (mg)	301.2	201.6	454.7

Laboratory test results were analyzed for each patient for the duration that the patient was in the study, i.e., until censoring or death. Median, mean, and standard deviations are displayed in Table 3. Median Hb was 116.1 g/L, MCH 30.7 pg, MCHC 322.8 g/L, serum iron 17.34 μmol/L, TSAT 29.76%, and serum ferritin 526.2 μg/L. Median Kt/V was 1.63, and median serum albumin 39.1 g/L. Median serum calcium was 2.35 mmol/l, serum phosphorus 1.76 mmol/L, calcium × phosphorus product 4.11 mmol^2^/L^2^, and intact PTH 301.4 ng/L.

**Table 3:** Patient median and mean values of tests analyzed during the total study duration

Laboratory test	Median	Mean	SD
Tests relevant to anemia			

Hemoglobin (g/L)	116.1	114.1	10.3
Hematocrit (Proportion of 1.0)	36.2	35.7	3.09
RBC (10^12/L)	3.76	3.76	0.4
MCV (fl)	94.6	94.6	7.14
MCH (pg)	30.7	30.5	2.54
MCHC (g/L)	322.8	322.3	12.7
Serum iron (μmol/L)	17.34	15.29	5.5
TSAT (%)	29.76	30.6	9.39
Total iron binding capacity (μmol/L)	42.46	44.05	10.41
Serum ferritin (μg/L)	526.2	593.9	426.76

Tests relevant to dialysis adequacy and nutrition			

Kt/V	1.63	1.61	0.27
Serum albumin (g/L)	39.1	38.7	4.2

Tests relevant to calcium and phosphorus metabolism			

Serum calcium (mmol/L)	2.35	2.34	0.17
Serum phosphorus (mmol/L)	1.76	1.81	0.57
Serum calcium × phosphorus (mmol^2^/L^2^)	4.11	4.2	1.07
Intact parathyroid hormone (ng/L)	301.4	385.1	330.21

### Univariate Analysis

**Figure 1:**
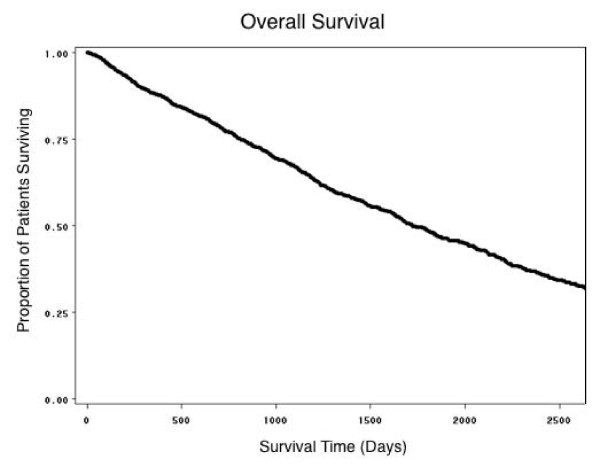
**Estimated proportion of patients surviving**.

**Figure 2:**
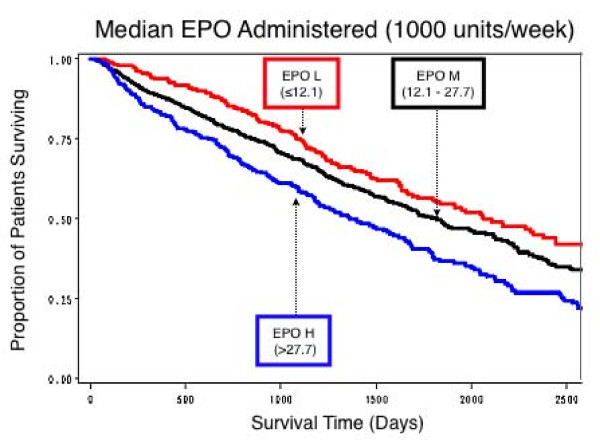
**Estimated proportion of patients surviving by levels of EPO administration**. Although the differences were small, survival was best in those receiving a low dose of EPO (L = ≤ 12.1 10^3 ^units per week), slightly worse with a medium dose (M = 12.1–27.7 10^3 ^units per week), and worst in those receiving a high dose (H = > 27.7 10^3 ^units per week).

**Figure 3:**
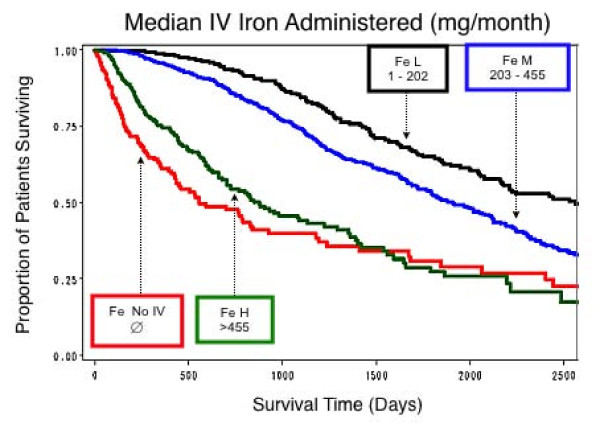
**Estimated proportion of patients surviving by levels of IV iron administration**. Survival was best in those receiving a low dose of IV iron (L = 1–202 mg per month), and slightly worse in those receiving a moderate dose (M = 202–455 mg per month). Survival was much worse in those who received a high dose (H = > 455 mg per month) and in those who did not receive IV iron (∅ = NO IV).

Survival curves were calculated for the patient group as a whole (Figure 1), for mean EPO per week and for mean IV iron per month of patient follow up. Patients were categorized into three groups (low, medium, high) based on the dose of each drug administered. In the case of IV Iron, patients who did not receive IV iron (No-IV) were also included along with the three other groups. Cut-offs for the various groups were based purely on the results of the statistical analysis (Table 2). For EPO, survival was relatively worse in the high dose group (> 27.74 10^3 ^units per week), and relatively best in the low dose group (≤ 12.10 10^3 ^units per week) (Figure 2). For IV iron, survival was worst in those who received no IV iron at all (Figure 3), and was almost as bad in the high dose group (≥ 455 mg/month). It was relatively best in patients receiving a relatively low dose (1–202 mg per month), slightly worse in those receiving a relatively higher dose (202.1–455 mg per month).

**Figure 4:**
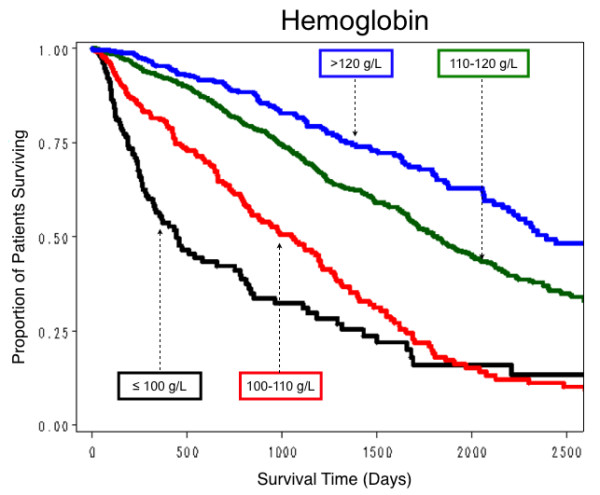
**Estimated proportion of patients surviving by four levels of hemoglobin**.

**Figure 5:**
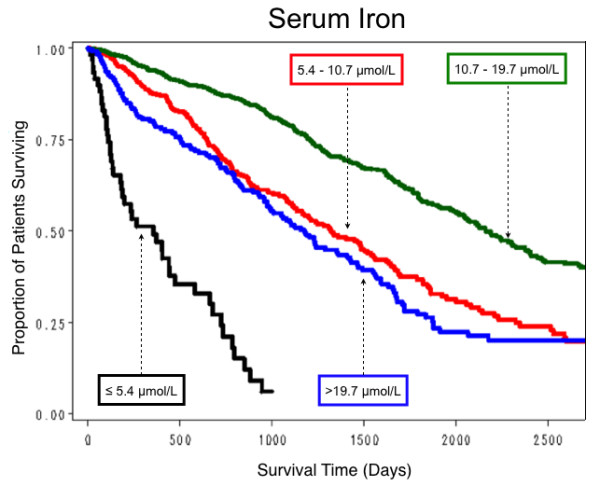
**Estimated proportion of patients surviving by four levels of serum iron**.

**Figure 6:**
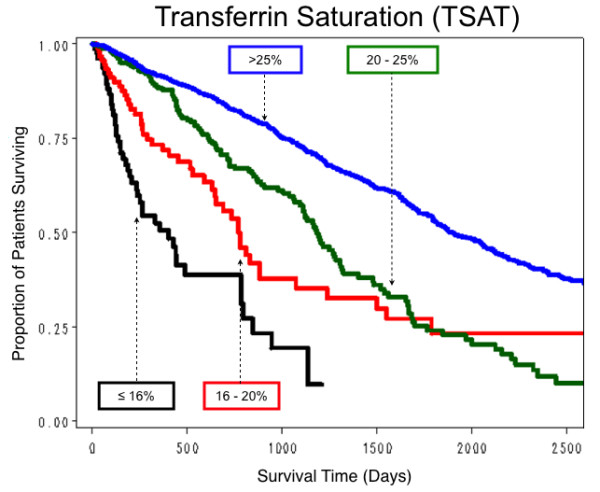
**Estimated proportion of patients surviving by four levels of TSAT**. The number of patients who received various levels of IV iron over the time at risk in each of the four TSAT levels is shown in Table 4.

**Figure 7:**
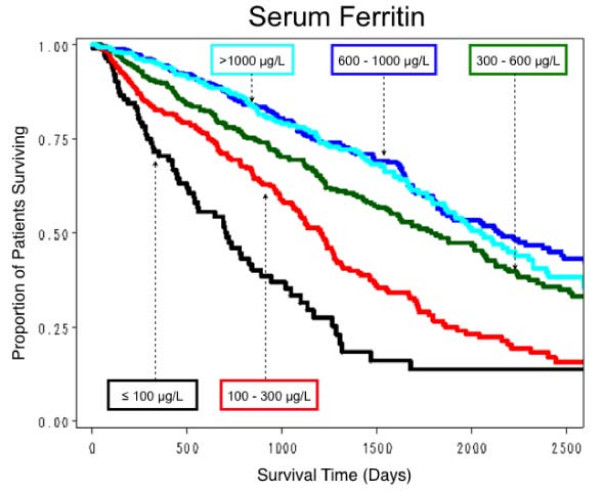
**Estimated proportion of patients surviving by five levels of serum ferritin**. The number of patients who received various levels of IV iron over the time at risk in each of the five serum ferritin levels is shown in Table 5.

All hematological parameters measured were quantitative but, to facilitate clinical interpretation, survival curves were obtained for selected clinically relevant categorizations of these parameter values. For Hb, survival was relatively the worst in those with an overall mean ≤ 100 g/L, and was progressively better with mean Hb levels of 100.1–110, 110.1–120, best in those with mean values > 120 g/L (Figure 4). For serum iron, survival was worst with a median serum iron level ≤ 5.4 μmol/L, best with a level 10.7–19.7 μmol/L (Figure 5). For TSAT, survival was worst with a mean TSAT ≤ 16%, and progressively better with mean TSAT levels 16.1–20, 20.1–25, and > 25% (Figure 6). No explicit relationship was observed between TSAT level and IV iron administered. For example, 17% of patients with TSAT levels ≤ 20% had received no IV iron, but 50% had received > 455 mg/month (Table 4). For serum ferritin survival was worst with mean serum ferritin ≤ 100 μg/L, slightly better with levels between 100 and 300 μg/L (Figure 7). No explicit relationship was observed between serum ferritin level and IV iron administered. For example, 40% of patients with serum ferritin levels > 600 μg/L had received IV iron > 455 mg/month but 18% had received no IV iron (Table 5). The best survival was in the three groups with mean serum ferritin levels between 300 and 600, 600 and 1000, and > 1000 μg/L, which differed little from each other. For Kt/V survival was worst with mean Kt/V ≤ 1.2, successively better with Kt/V 1.21–1.4, 1.41–1.6, and best with Kt/V > 1.6. For serum albumin, median survival was worst with a mean value ≤ 35 g/L, best with a mean value > 40 g/L.

**Table 4:** The number of patients who received various levels of IV iron over the time at risk in each of the four TSAT levels

TSAT level	IV iron level (mg/month)			
	
	0	1 – 202	202.1 – 455	> 455
≤ 16%	16	3	16	45
16.1 – 20%	14	6	30	41
20.1 – 25%	20	18	142	91
> 25%	126	219	695	263

**Table 5:** The number of patients who received various levels of IV iron over the time at risk in each of the five serum ferritin levels

Serum ferritin level	IV iron level (mg/month)			
	
	0	1 – 202	202.1 – 455	> 455
≤ 100 μg/L	30	23	34	34
100.1 – 300 μg/L	37	75	162	124
300.1 – 600 μg/L	31	65	233	123
600.1 – 1000 μg/L	34	52	291	161
> 1000 μg/L	29	28	161	53

### Multivariate analysis

Survival duration was first analyzed using data for all years 1998–2006 for all patients in all 3 dialysis units. Included in the model were age at treatment start, race, gender, presence or absence of co-morbid conditions, EPO administration in 3 ranks (0–12.1, 12.11–27.7, and > 27.7 10^3 ^units per week), IV iron administration in 4 ranks (0, 1–202, 202–455, and > 405 mg per month), and median values for all laboratory test results. Race, gender, dialysis center, time of entry into the study, serum iron, TIBC, MCV, MCH, serum calcium, and serum phosphorus were eliminated from the model in backward elimination as they failed to reach the level of significance (P < 0.05). The results, summarized in part in Table 6, demonstrate the powerful independent positive effect on survival of intravenous iron administered in low (≤ 202 mg/month) and medium (202–455 mg/month) doses. TSAT levels > 25% and serum ferritin levels > 300 μg/L were also independently associated with a positive effect on survival. Serum albumin > 40 g/L and Kt/V > 1.6, a measure of dialysis adequacy, were associated with a positive effect on survival, as has long been recognized [20]. TIBC, MCHC, calcium × phosphorus product, and PTH were factors that also independently affected survival.

**Table 6:** Cox Regression: results of a model in which all data for all units are included.

Factors analyzed	Reference Value*	Hazard Ratio (HR)	HR 95% Confidence Limits
Age at treatment start (per year)		1.045	1.04, 1.05
Co-morbid conditions (None)	≥ 1	0.72	0.60, 0.87
EPO 0–12.1 10^3 ^units/week	> 27.7 10^3 ^units/week	1.5	1.14, 1.96
EPO 12.1 – 27.7 10^3 ^units/week	> 27.7 10^3 ^units/week	1.35	1.10, 1.67
IV iron 0 mg/month	> 455 mg/month	1.27	0.91, 1.77
IV iron 1 – 202 mg/month	> 455 mg/month	0.27	0.20, 0.36
IV iron 202 – 455 mg/month	> 455 mg/month	0.49	0.69, 0.61
Hb ≤ 100 g/L	> 120 g/L	2.83	1.92, 4.16
Hb 100.1 – 110 g/L	> 120 g/L	2.69	1.98, 3.67
Hb 110.1 – 120 g/L	> 120 g/L	1.67	1.31, 2.14
TSAT ≤ 16%	> 25%	1.4	0.92, 2.10
TSAT 16.01 – 20%	> 25%	1.33	0.94, 1.88
TSAT 20.01 - 20%	> 25%	1.41	1.14, 1.76
Serum ferritin ≤ 100 μg/L	> 1000 μg/L	3.64	2.49, 5.32
Serum ferritin 101–300 μg/L	> 1000 μg/L	2.2	1.66, 2.90
Serum ferritin 301–600 μg/L	> 1000 μg/L	1.45	1.11, 1.89
Serum ferritin 601–1000 μg/L	> 1000 μg/L	1.14	0.88, 1.49
Serum albumin ≤ 35 g/L	> 40 g/L	3.41	2.58, 4.50
Serum albumin 35.1–40 g/L	> 40 g/L	1.28	1.05, 1.57
Kt/V ≤ 1.2	> 1.6	2.18	1.56, 3.05
Kt/V 1.21-1.4	> 1.6	1.37	1.03, 1.73
Kt/V 1.41-1.6	> 1.6	0.87	0.70, 1.07

* The numbers of patients in the reference groups are below, in parenthesis: EPO > 27.7 10^3 ^units/week (n = 400); IV iron > 455 mg/month (n = 442); Hb > 120 g/L (n = 442); TSAT > 25% (n = 1303); serum ferritin > 1000 μg/L (n = 266); serum albumin > 40 g/L (n = 688); Kt/V > 1.6 (n = 950)

### Analysis of the EPO IV iron interaction effect on patient survival

**Figure 8:**
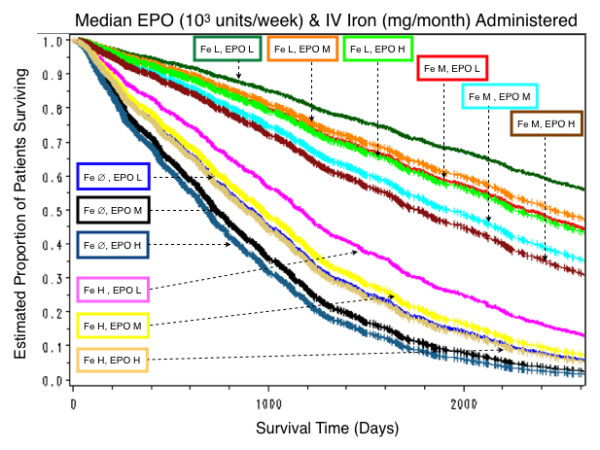
**Estimated proportion of patients surviving by combinations of levels of IV iron and of EPO administration**. Low (L), medium (M) and high (H) levels of iron and EPO administered are indicated in the boxes by the appropriate symbol, and those who received no iron by the symbol ∅. The worst survival estimates were in patients who received no iron (Fe ∅). Survival estimates were slightly, but little better in those in the high iron group (Fe H). the best survival estimates were in the low iron group (Fe L), and they were slightly worse in the medium iron group (Fe M). For each of the four levels of IV iron administered, survival was best in the low EPO Group (EPO L), and worst in the high EPO group (EPO H), but the differences were very small.

This analysis was done with a model in which only IV iron administered and EPO administered were included as interactions along with all the other significant predictors of survival. The results are summarized in Table 7. Estimated survival curves are displayed in Figure 8. For IV iron, survival was uniformly worst for those who received no IV iron, best with 1–202 mg/month, and slightly worse with 202–455 mg/month. For those who received IV iron > 455 mg/month, survival was only slightly better than in the no IV iron group. The effect of EPO on survival was weak but had statistically significant interaction with IV iron. In particular, EPO without IV iron did not seem to have any beneficial impact on survival, whereas with IV iron EPO did have some beneficial effect on survival, albeit small.

**Table 7:** IV iron EPO administered interaction: hazard ratios and patient survival

Interaction	Patients (n)	IV Iron (mg/month)	EPO (10^3 ^u/week)	Hazard	HR 95% Confidence Limits	Median Survival (days)
A	21	0	0–12.1	0.82	0.37, 1.83	317
B	89	1–202	0–12.1	0.45	0.28, 0.73	1623
C	238	202.1–455	0–12.1	0.86	0.60, 1.23	1104
D	83	>455	0–12.1	1.37	0.81, 2.32	314
E	80	0	12.1–27.7	3.12	2.02, 4.81	152.5
F	124	1–202	12.1–27.7	0.35	0.23, 0.52	1672
G	458	202.1–455	12.1–27.7	0.68	0.51, 0.92	993
H	200	> 455	12.1–27.7	1.6	1.11, 2.31	331.5
I	53	0	> 27.7	1.27	0.72, 2.24	282
J	32	1–202	> 27.7	0.41	0.24, 0.71	1247.5
K	187	202.1–455	> 27.7	0.52	0.38, 0.72	985
L	158	> 455	> 27.7			620.5

### Analysis of the TSAT EPO interaction effect on patient survival

**Figure 9:**
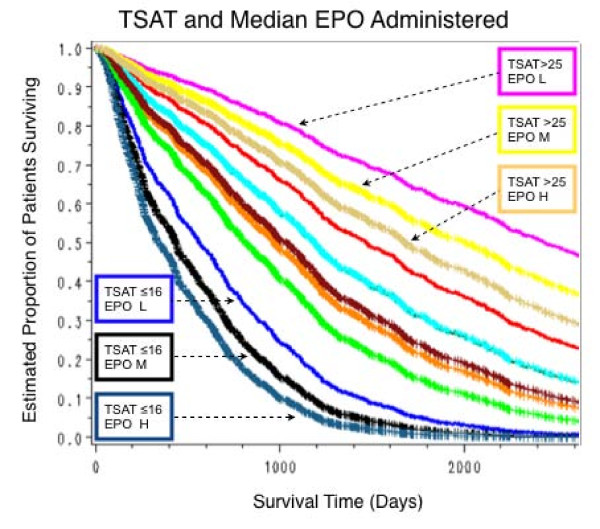
**Estimated proportion of patients surviving by combinations of four levels of TSAT and three of EPO administration**. TSAT levels are shown in the boxes and low (L), medium (M) and high (H) levels of EPO administered are indicated by the appropriate symbol. The 3 worst survival estimates were in patients with TSAT ≤ 16%; of the three, the best estimate was for the low EPO (L) group. The three best survival estimates were in patients with TSAT > 25%; of the three, the best was for the low EPO (L) group. Estimates for intermediate levels of each variable are shown but not identified specifically. Survival in patients with TSAT 16.01–20% was better than that of those with TSAT ≤ 16%; in those with TSAT 20.01–25% it was better than in those with TSAT 16.01–20%, and worse than in those with TSAT > 25%. For each of these two TSAT levels, as well as for the two other TSAT levels, survival was best in the low EPO (L) group, and worst in those in the high (H) EPO group.

This analysis was done with a model in which only TSAT and EPO administered were included as interactions along with other significant predictors of survival. As compared with patients with a mean TSAT > 25%, the hazard ratios for those with TSAT ≤ 16% was 6.62, for TSAT 16.01–20 it was 2.59, and for TSAT 20.01–25 it was 1.95. As compared with patients who received EPO > 27.7 10^3 ^units/week the hazard ratios for those who received 0–12.1 and 12.1–27.7 10^3 ^units/week were, respectively, 0.61 and 0.81. An interaction of TSAT and EPO administration on survival was not significant. The estimated survival curves (Figure 9), demonstrate the powerful effect of low TSAT levels on survival even after controlling for other significant predictors of survival.

### Analysis of the serum ferritin EPO interaction effect on patient survival

This analysis was done with a model in which only serum ferritin and EPO administered were included as interaction along with other significant predictors of survival. The results are summarized in Table 8. There was a significant adverse effect on survival of a serum ferritin level ≤ 100 μg/L at all three levels of EPO administration (0–12.1, 12.11–27.7 10^3 ^units/week), i.e, interactions A, F, and K; the hazard ratios were, respectively, 7.37, 5.93, and 3.39. The adverse effect on survival was still apparent but less marked when serum ferritin levels between 100 and 300 μg/L were considered (interactions B, G, and L). When higher serum ferritin levels were considered (between 300 and 600, 600 and 1000 and > 1000 μg/L), with a single exception (Interaction M) no statistically adverse effects on survival were observed.

**Table 8:** Serum ferritin EPO administered interaction: hazard ratios and patient survival

Interaction	Patients (n)	Serum ferritin (μg/L)	EPO given (10^3 ^u/week)	Hazard Ratio (HR)	HR 95% Confidence Limits	Median Survival (days)
A	24	≤ 100	0–12.1	7.37	3.40, 16.0	224
B	85	101–300	0–12.1	4.14	2.24, 7.67	569
C	110	301–600	0–12.1	2.16	1.18, 3.94	956
D	127	601–1,000	0–12.1	1.59	0.87, 2.92	1104
E	80	> 1,000	0–12.1	1.89	1.04, 3.41	1278
F	58	≤ 100	12.1–27.7	5.93	3.23, 10.90	492
G	198	101–300	12.1–27.7	3.79	2.23, 6.43	507
H	231	301–600	12.1–27.7	2.24	1.33, 3.78	715
I	229	601–1,000	12.1–27.7	1.73	1.01, 2.94	1111
J	131	> 1,000	12.1–27.7	1.67	0.96, 2.89	1189
K	34	≤ 100	> 27.7	3.39	1.61, 7.14	303
L	110	101–300	> 27.7	2.27	1.33, 3.90	621
M	106	301–600	> 27.7	2.1	1.202, 3.62	604
N	115	601–1,000	> 27.7	1.50	0.90, 2.67	784
O	52	> 1,000	> 27.7			934

## Discussion

### Hemoglobin

In a recent study of almost 160,000 HD patients who had Medicare as their primary payer both highly variable and persistently and transiently low Hb levels (< 110 g/L) in the first 6 months of 2004 were associated with increased risk of death in the second six months of the same year, whereas transiently and persistently high Hb levels (> 125 g/L or > 120 g/L) were not [21].

Taking confounding factors and other significant predictors of survival into account, our long-term observations showed clearly that patient survival was better the higher the Hb, and that survival was best with a Hb level > 120 g/L. The 50% survival estimates for Hb > 120, 110–120, 100–110, and ≤ 100 g/L were, respectively, 1198, 1144, 751, and 534 days.

Recent controlled clinical trials that reported adverse effects of a high Hb level in CKD patients [2,3] are the basis for warnings about adverse effects of raising Hb above 120 g/L. The trials were of short duration, without reference to the iron status of the patients or the known iron deficiency of CKD patients [6-9], and were designed to increase the Hb concentration by manipulating the ESA dose. Moreover, supplementary iron was not provided to patients whose relatively dormant bone marrow would have been stimulated by the ESA, thereby depleting iron stores available for metabolic functions other than Hb production [2,3,9,10].

The development of thrombocytosis is one possible mechanism for adverse effects of ESAs in the presence of depleted iron stores. An inverse relationship between TSAT and platelet counts and platelet volume has been demonstrated in women with iron deficiency anemia [22]. Evidence has recently been presented suggesting that, in HD patients given high doses of EPO to achieve Hb ≧ 130 g/L, iron depletion and the associated relative thrombocytosis might play a role in the reported increased mortality [23].

Studies on cytokines in chronic HD patients point to another possible mechanism for adverse effects on morbidity and mortality of EPO administration in iron deficient or marginally iron sufficient patients. The pro-inflammatory cytokine TNF-α was reported to be about 6 times higher in HD patient plasma than in controls (5.6 ± 0.9 versus 0.9 ± 0.1 pg/ml) [24]. In another study, plasma concentrations of TNF-α, the anti-inflammatory cytokine IL-4, and total peroxide concentrations were measured in a 90-day randomized controlled trial in patients receiving EPO without or in combination with IV iron [25]. Patients receiving both IV iron and EPO had lower pro-inflammatory cytokine TNFa and higher anti-inflammatory cytokine IL-4 levels, as well as lower levels of total peroxide a marker of free radical concentration.

These observations suggest a possible role for these cytokines in relation to the adverse effect of iron deficiency on morbidity and mortality in EPO treated CKD and HD patients in whom there was inattention to the iron status.

### Non-hematological effects of iron deficiency

The renal literature refers rarely to non-hematological consequences of iron deficiency. Iron is a key component of many cellular enzymes, including oxidases, catalases, peroxidases, aconitases, and nitric oxide synthetases [26-28]. Iron deficiency adversely affects metabolic processes that include electron transport, catecholamine metabolism, DNA synthesis, and several enzyme systems [4]. Iron is an integral part of the requirement of mitochondrial enzymes of the electron transport chain, and of cytochromes and iron-sulfur proteins required for oxidative phosphorylation of ADP to ATP, and iron deficiency in rats leads to oxidant-induced mitochondrial damage [29]. Iron deficiency results in decreased exercise performance in rats, corrected rapidly by iron but not by blood transfusion [30,31]. In HD patients iron deficiency without evidence of iron deficiency anemia has been reported, and IV iron repletion was associated with positive effects on serum albumin, muscle mass, hospitalization, and blood pressure [32]. Low serum iron (< 8.1 μmol/L) and low TSAT levels were shown to be associated with increased mortality [33]. In the present study survival was worst with very low levels of serum iron (≤ 5.4 μmol/L), TSAT (≤ 20%), and serum ferritin (≤ 100 μg/L), and in those who received no IV iron.

When ESAs are administered to patients deficient or marginally sufficient in iron, the demands for iron for Hb production are likely to further deplete iron available for other essential metabolic processes The rise in plasma TNF-α and decrease in IL-4 levels may also be important as noted above. We conclude that close attention to iron repletion and iron sufficiency is essential for HD patient well-being.

### Seeking an optimal dose of IV iron for hemodialysis patients

Widely publicized clinical guidelines caution about administering IV iron to HD patients with serum ferritin levels above thresholds varying from 500 to 800 μg/L [14-16,34], but recent reviews have concluded that an evidence based guideline for an upper limit of serum ferritin is not available [35,36]. Ferritin levels up to 1200 μg/L were not associated, in a large study, with an increase in all cause mortality [37]. The present study confirms this by demonstrating clearly that serum ferritin levels of between 600 and 1,000 and > 1,000 μg/L were not associated with adverse effects on survival in univariate and multivariate analysis. There is no clear evidence for the association of infection with high serum ferritin levels in dialysis patients [38-40]. We found that whereas malnutrition and infections are, not surprisingly, associated with patient deaths, neither a serum ferritin level > 600 μg/L nor a high dose of iron (> 455 mg/month) was significantly associated with either infection or malnutrition (Table 9). That the best survival was observed in patients with a serum iron level > 10.7 μmol/L and with a TSAT level > 25% also confirms the report of Kalantar-Zadeh and colleagues [37].

**Table 9:** Outcomes, serum ferritin levels, and IV iron administration in relation to malnutrition and certain infections

Disease	Outcome				Serum Ferritin Level				IV iron			
(ICD9-CM)	Died	Alive	χ^2^[1]	p	< 600 (μg/L)	> 600 (μg/L)	χ^2^[1]	p	≤ 455 (mg/mo)	> 455 (mg/mo)	χ^2^[1]	p

Patients (n)	600	465			698	720			810	318		
Malnutrition (ICD 262–263)	73	40	44.9	< 0.001	47	66	2.54	0.11	78	19	0.32	0.85
Infection (ICD 001–139)	250	418	2.42	< 0.12	322	346	0.07	0.79	398	143	1.43	0.23
Pneumonia (ICD 480–486)	143	127	44.8	< 0.001	141	127	1.35	0.24	183	48	0.01	0.92
Cellulitis & carbuncle (ICD 680–682)	97	86	28.1	< 0.001	86	97	0.32	0.57	137	40	2.98	0.08
Ulcer of skin (ICD 707)	102	56	65.4	< 0.001	77	81	0	1	124	38	1.83	0.17

Survival decreases sharply as serum iron and TSAT levels decrease in both univariate and multivariate analyses: this strongly suggests that serum iron > 10.7 μmol/L and TSAT > 25% are reasonable targets to assess iron sufficiency in HD patients. When the needed amount of IV iron is considered in relation to these targets and survival our data showed that amounts up to 455 mg per month were associated with improved survival. On the other hand, amounts > 455 mg per month, close to the reported cut-off point of 400 mg per month reported by Kalantar-Zadeh and colleagues [37], were associated with worsened survival.

In this study we summarized hematological parameters and rates of administration of EPO and IV iron for each patient to assess the effects of these parameters and treatments on survival. Assessing effects of temporal changes in treatments or hematological parameters on patient's survival is also important and is subject of future investigation.

## Conclusion

• In HD patients a high Hb level (> 120 g/L) was associated with the best survival, a relatively low Hb level (≤ 110 g/L) with the worst.

• With respect to parameters reflecting iron status, the best survival was associated with a high serum iron (> 10.7 μmol/L), TSAT (> 25%), and serum ferritin (> 600 μg/L), the worst with low levels for all three measurements.

• The influence of IV iron on survival was profound. The best survival was in patients receiving 1–202 mg per month, the worst in patients receiving no IV iron at all, and in those receiving > 455 mg per month.

• The influence of EPO on survival was very small, but the best survival was in patients receiving EPO ≤ 12.1 10^3 ^units per week.

## Competing interests

Victor E. Pollak is Founder, Member of the Board of Directors, owner of stock, and full-time employee of MIQS, Inc.

Jonathan A. Lorch is owner of stock of MIQS, Inc.

## Authors' contributions

VEP designed the medical content of the database, designed and implemented the study (with JAL) and interpreted the data (with JAL and RS). He drafted and revised the manuscript. JAL implemented the software in the 3 dialysis units that are the subjects of the study, acquired the data and designed and implemented the study (with VEP) and revised the manuscript. RS designed the statistical analysis and interpretation, and was involved in revising the manuscript. SS was involved in the statistical analysis of the data. All authors read and approved the final manuscript.

## Pre-publication history

The pre-publication history for this paper can be accessed here:

http://www.biomedcentral.com/1471-2369/10/6/prepub
